# Application of species-specific primers to estimate the *in situ* diet of *Bythotrephes* [Cladocera, Onychopoda] in its native European range via molecular gut content analysis

**DOI:** 10.1093/plankt/fbab070

**Published:** 2021-10-28

**Authors:** Arthur Pichler, Tina L Walters, Marc E Frischer, Jens C Nejstgaard, Radka Ptáčníková

**Affiliations:** WasserCluster Lunz – Biological Station GmbH, Dr. Carl Kupelwieser Promenade 5, 3293 Lunz am See, Austria; University of Vienna, Department of Functional and Evolutionary Ecology, Althanstraße 14, 1090 Vienna, Austria; University of Georgia, Skidaway Institute of Oceanography, 10 Ocean Science Circle, Savannah, GA 31411, USA; University of Georgia, Skidaway Institute of Oceanography, 10 Ocean Science Circle, Savannah, GA 31411, USA; Department of Experimental Limnology, Leibniz-Institute of Freshwater Ecology and Inland Fisheries, Zur alten Fischerhütte 2, D-16775 Stechlin, Germany; WasserCluster Lunz – Biological Station GmbH, Dr. Carl Kupelwieser Promenade 5, 3293 Lunz am See, Austria

**Keywords:** spiny water flea, zooplankton, trophic interactions, perialpine lakes, invasive species

## Abstract

The study of invasive species often focuses on regions of recent introduction rather than native habitats. Understanding an invasive species in its natural environment, however, can provide important insights regarding the long-term outcome of invasions. In this study we investigated the diet of the invasive spiny water flea, *Bythotrephes longimanus*, in two Austrian perialpine lakes, where it is native. The gut contents of wild-caught *Bythotrephes* individuals were estimated by quantitative polymerase chain reaction, targeting species-specific fragments of the barcoding region of the cytochrome c oxidase I gene of potential prey. The observed prey spectrum of *Bythotrephes* in the study lakes consisted primarily of *Eudiaptomus gracilis* and *Diaphanosoma brachyurum*. The *Daphnia longispina* complex, *Leptodora kindtii* and *Mesocyclops leuckarti* also contributed to the diet. Results indicate that *Bythotrephes* is a generalist feeder with a preference for epilimnetic prey.

## INTRODUCTION

Biological invasions are recognized as important driver of global environmental change (Carpenter *et al.*, 2011). Freshwater ecosystems are especially vulnerable to invasions and its effects (Sala *et al.*, 2000). Although at least conspicuous invasive species with implicit impacts in their new environment are relatively well studied, detailed knowledge of the same species in their native areas is often lacking. Thus, there has been a continued bias toward studying invaders primarily in their introduced ranges (Parker *et al.*, 2013). Among other impacts, invasives may affect existing trophic relationships, which, as key mediators of ecosystem functioning, determine community dynamics (Cardinale *et al.*, 2012). Understanding the diet of invasives is a crucial factor in understanding biological invasions. Diet studies of both invasive and native populations are imperative to understand dietary requirements, dietary flexibility and thus potential impacts of invasive species (Courant *et al.*, 2017). For instance, a broad dietary spectrum is a frequently cited characteristic of invasives, yet successful invaders do not necessarily exhibit a broad diet (Vázques, 2006).

The spiny water flea, *Bythotrephes* Leydig 1860, is a genus of predatory freshwater cladocerans with an extensive Eurasian native distribution. It occurs around the Central European Alps, in Northern Europe and throughout countries of the former Soviet Union into China (Therriault *et al.*, 2002). *Bythotrephes* gained considerable attention after invading the Laurentian Great Lakes in North America in the 1980s (Sprules *et al.*, 1990). Since then it has established in numerous lakes in Canada and the USA (e.g. Yan *et al.*, 1992; Kerfoot *et al.*, 2016), subsequently followed by decreases in species richness (Yan *et al.*, 2002; Barbiero and Tuchman, 2004; Kelly *et al.*, 2013) and biomass of zooplankton (Kerfoot *et al.*, 2016). In contrast, other zooplankton species increased to previously unrecorded levels (Yan and Pawson, 1997). In European lakes with *Bythotrephes*, the observed species diversity of zooplankton was unaffected or even higher than in similar lakes where no *Bythotrephes* were found (Hessen *et al.*, 2011; Kelly *et al.*, 2013; Walseng *et al.*, 2015; Horváth *et al.*, 2017). However, the predation pressure exerted by *Bythotrephes* in Lago Maggiore, Italy, was deemed high enough to seasonally reduce *Daphnia* densities to low values (Manca *et al.*, 2008). Additionally, the observed long-term rise in water temperature and changes in the thermal stratification of Lago Maggiore were assumed to positively affect reproduction and population density of native *Bythotrephes* and thus be associated with dramatic changes in the pelagic food web (Manca *et al.*, 2007). These results further strengthen the need to study *Bythotrephes* in its native habitat.

*Bythotrephes* is largely considered a generalist predator. In some experimental feeding studies, cladocerans, including *Daphnia*, *Ceriodaphnia* and *(Eu)Bosmina*, were strongly preferred prey for *Bythotrephes*, whereas in other studies calanoid and cyclopoid copepodites, nauplii and even adult copepods (*Tropocyclops extensus*) were common prey (Vanderploeg *et al.*, 1993; Dumitru *et al.*, 2001; Wahlström and Westman, 2011; Jokela *et al.*, 2013). Some feeding studies observed a more specialized predation. Despite offering several prey species (*Daphnia mendotae, B. longirostris, Diaptomus sp.* and *Diacyclops thomasi*), Pangle and Peacor (2009) only observed the consumption of *D. mendotae*. In a similar experiment, *Bythotrephes* reduced the abundance of *Daphnia*, *Bosmina* and *Ceriodaphnia*, whereas copepods and *Diaphanosoma* were not preyed upon (Jokela *et al.*, 2013). However, laboratory feeding studies can be difficult to interpret and it is recognized that experimentally measured responses to prey, e.g. prey choice, may not accurately reflect responses in the field (Van Lenteren and Bakker, 1975; Symondson *et al.*, 2002; McKemey *et al.*, 2003). Allozyme analyses of wild-caught *Bythotrephes* from Lake Michigan (Schulz and Yurista, 1995) indicated a diverse diet consisting of cladocerans, cyclopoid copepods and calanoid copepods.

The use of Molecular Gut Content Analysis (MGCA) approaches has increased the precision of diet estimations due to high taxonomic resolution and sensitivity to macerated or degraded prey tissue (King *et al.*, 2008; Nielsen *et al.*, 2018). DNA-based methods of diet estimation compare well to known (fed) diet when there is a small number of potential resources, but similarity between different methods of diet estimation generally declines with increasing number of potential resources (Nielsen *et al.*, 2018). Quantitative polymerase chain reaction (qPCR)-based techniques have successfully been implemented in zooplankton diet studies: from laboratory feeding experiments revealing the first molecular detection of a specific species of algae in calanoid copepod guts and fecal matter (Nejstgaard *et al.*, 2003) and the detection of predation of *Acartia nauplii* in calanoid copepods (Durbin *et al.*, 2008), to *in situ* diet studies of wild-caught doliolids (Frischer *et al.*, 2014; Walters *et al.*, 2019). In their studies on copepods both Nejstgaard *et al.* (2008) and Durbin *et al.* (2008) noted that the amount of DNA estimated via qPCR was lower than expected. The possibility of rapid digestion resulting in an underestimation of stomach contents was later confirmed (Simonelli *et al.* 2009; Troedsson *et al.* 2009; Durbin *et al.* 2012). The high sensitivity of DNA-based diet analyses also comes with the disadvantage of possible detection of false positives due to contamination, which is an ubiquitous problem, especially challenging in small aquatic invertebrates due to the surrounding water teaming with dietary components (Passmore *et al.*, 2006; King *et al.*, 2008). In qPCR assays, the cycle threshold (ct) value is defined as the number of cycles required for the fluorescent signal to exceed background levels. Hence, samples with high amounts of target DNA require fewer cycles than samples with little target DNA. It is a common approach, especially in clinical studies and zoonotic disease research, to apply a cutoff of late ct values and only regard results with fewer cycles as true positives (e.g. Caraguel *et al.*, 2011; Baerwald *et al.*, 2012; Bolotin *et al.*, 2015; Aminu *et al.*, 2020).

In this study, we estimated the *in situ* diet of *Bythotrephes* using qPCR. We designed primer pairs targeting short sequences of the mitochondrial cytochrome c oxidase subunit I (COI) gene of potential crustacean prey species, occurring in two study lakes of the native region of *Bythotrephes longimanus*, Erlaufsee and Mondsee, located in the Austrian perialpine region. The tested prey species included common taxa of oligo- and mesotrophic European lakes: the cladocerans *Daphnia longispina* complex, *(Eu)bosmina* sp., *Leptodora kindtii* and *Diaphanosoma brachyurum*, and the copepods *Eudiaptomus gracilis*, *Mesocyclops leuckarti*, *Cyclops vicinus* and *Cyclops sp.* X (see Method). Our aim was to design and apply species-specific primers for potential crustacean prey species in the study lakes and to provide an estimation of the *in situ* diet of *Bythotrephes* in its native range to elucidate its choice of prey in the field and improve the understanding of the trophic role of this predator in lake food webs.

## METHOD

### Sampling

Zooplankton, including *Bythotrephes*, were collected during the day (10 a.m.–5 p.m.) via vertical tows from the same locations from the open water of the perialpine lakes Mondsee (47°50′43.3″N, 13°21′12.0″E, sampling location: 30 m depth) and Erlaufsee (47°47′29.2″N, 15°16′29.1″E, sampling location: deepest point; 38 m depth), Austria. Zooplankton communities of both lakes are representative of perialpine lakes in Austria and Germany (Horváth *et al.*, 2017). The lakes were sampled biweekly between May and November 2018, using closable nets to sample epilimnion, metalimnion and hypolimnion, and net mesh sizes of 40 μm, 100 μm and 285 μm. For abundance estimates zooplankton was subsampled prior to counting. Entire samples were examined for *Bythotrephes* and *Leptodora*. *Bythotrephes* used in qPCR were collected across the season (Table I) to minimize bias of one-time sampling. Samples were fixed and stored in 80% ethanol. Zooplankton specimens were separated and rinsed in ethanol before DNA extraction.

**Table I:** Number, sampling dates and sampling depths of *Bythotrephes* specimens analyzed by qPCR

	Date	Specimens	Depth	EG	DA	LK	BO	DB	ML	CX
Mondsee	19.6.	10	0–25 m	3/4/3	1/3/6	0/6/4	-	1/5/4	-	0/2/8
	18.7.	10	0–15 m	0/1/9	1/3/6	0/3/7	-	0/2/8	-	0/0/10
	18.8.	10	4–15 m	7/3/0	4/5/1	1/9/0	0/0/7	3/0/0	1/5/1	0/2/8
	29.8.	10	0–15 m	7/1/2	1/2/7	1/5/4	0/0/10	9/1/0	3/5/2	0/5/4
	17.9.	10	0–15 m	1/4/5	0/0/10	2/8/0	0/0/10	-	1/2/7	0/0/10
	22.10.	10	0–15 m	1/2/7	0/1/9	0/1/9	0/2/8	0/3/1	0/5/5	0/1/9
	13.11.	10	0–15 m	2/2/6	0/0/10	0/2/8	0/1/9	0/2/8	0/5/5	0/1/9
	Total	70		21/17/32	7/14/49	4/34/32	0/3/44	13/13/21	5/22/20	0/11/58
Erlaufsee	16.7.	6	0–15 m	1/2/3	1/1/4	0/4/2	0/0/6			
	17.8.	10	0–15 m	6/4/0	2/5/3	0/8/2	0/3/7			
	30.8.	9	0–15 m	5/3/1	2/2/5	0/6/3	0/1/5			
	18.9.	8	0–15 m	1/7/0	4/2/2	0/7/1	0/0/8			
	23.10.	10	0–15 m	1/1/8	2/3/5	0/6/4	-			
	14.11.	10	0–15 m	0/4/6	1/4/5	3/6/1	-			
	Total	53		14/21/18	12/17/24	3/37/13	0/4/26			

Results for each prey species show: number of positive specimens (ct < 36)/number of inconclusive specimens (ct = 36–40)/number of negative specimens [EG: *E. gracilis*, DA: *Daphnia*, LK: *Leptodora kindtii*, BO: *Bosmina*, DB: *Diaphanosoma brachyurum*, ML: *M. leuckarti*, CX: *Cyclops sp. X*].

### DNA extraction

Total DNA was extracted from whole individuals of *Bythotrephes* and potential prey species using the QIAGEN DNeasy Blood & Tissue Kit (Valencia, CA, USA) following manufacturer protocols. DNA was eluted in 100 μL of AE buffer and stored at −20°C. Following genomic DNA (gDNA) extraction, purified DNA was quantified using a Qubit 2.0 Fluorometer (Invitrogen, Carlsbad, CA, USA) and a Qubit dsDNA High Sensitivity Assay Kit (Life Technologies, Eugene, OR, USA). Amenability of the extracts for PCR amplification was determined by amplifying 18S rRNA using universal primers Univ-18SF-577F (5′ CAG CAG CCG CGG TAA TTC C 3′) and Univ 18S-1180R (5′ CCC GTG TTG AGT CAA AAG C 3′) as previously described (Frischer *et al.*, 2017). Amplification was accomplished using a GeneAmp 9 700 Thermocycler (Applied Biosystems, Foster City, CA) and included a 3 min initial denaturation at 95°C followed by 30 amplification cycles [94°C (30 s), annealing temperature of 60°C for 30 s, 72°C (1 min)] followed by a 10 min final extension step at 72°C.

### Prey species and primer design

A set of 8 primer pairs (Table II) targeting short (<200 bp) fragments of the mitochondrial COI was designed for the cladocerans *D. longispina* complex (represented by *D. longispina* and *Daphnia cucullata* in Mondsee and *D. longispina* in Erlaufsee, hereinafter referred to as *Daphnia*), *Bosmina* (represented by *Bosmina (Eubosmina) coregoni* and *Bosmina longirostris)*, *L. kindtii* and *D. brachyurum*, and the copepods *E. gracilis*, *Mesocyclops leuckarti*, *C. vicinus* and *Cyclops sp.* X*. Daphnia*, *Bosmina, L. kindtii* and *E. gracilis* occurred in both of the lakes. *M. leuckarti*, *D. brachyurum* and *Cyclops sp.* X were present in Mondsee only. *Cyclops sp.* X (Krajíček *et al.*, 2016) is a, formally undescribed, European cyclopoid copepod, often misidentified as *Cyclops abyssorum*. *C. vicinus*, while present in Erlaufsee, was not included in the final qPCR analyses, because it was extremely rare in our zooplankton samples.

**Table II:** Species-specific primer pairs designed for this study

*Species*	*Primer*	*Sequence (5′–3′)*	*Expected product size (bp)*	*Annealing temperature (°C)*
*M. leuckarti*	COI_3197F	GGTAATATGCGGACCTTGGG	135	55
	COI_3312R	TCGGTCAGTCAATAATATGGTGA		
*E. gracilis*	COI_3071F	CTCTCTAGGAACATCGCGCA	89	56
	COI_3145R	GCCCCTAGAATAGACCTAACCC		
*Leptodora kindtii*	COI_3066F	CCCCTCTTTCAGCTGCAATC	142	56
	COI_3203R	GTCATTCCTGTTGAGCGCAT		
*Diaphanosoma brachyurum*	COI_2986F	CCCCTTCTCTGACCCTTCTT	72	56
	COI_3039R	GGTAGACAGTTCAGCCGGT		
*C. vicinus*	COI_N222F	CTCGTCCCTTTGATGCTTGG	138	56
	COI_N341R	TGTTCATCCAGTCCCTGCC		
*Cyclops sp. X*	COI_N212F	CGGAAATTGGTTAGTGCCCC	135	56
	COI_N327R	CCAGCCCCTCTTTCTACCAA		
*Bosmina*	COI_N370F	CTCTCTCGTCAGGACTAGGTC	145	61
	COI_N495R	TCTAACGACAGACCTTCCCC		
*Daphnia*	COI_N83F	ACTAATCCGGGCTGAACTTG	80	61
	COI_N141R	CGTGGGCAGTTACAATTACATT		

Amplification included a 3 min initial denaturation at 95°C followed by 30 amplification cycles [94°C (30 s), annealing temperature (see above) for 30 s, 72°C (30 s)] and a 10 min final extension step at 72°C.

To facilitate primer design, COI representative sequences for targeted prey species were obtained from Genbank. In the case where sequences of target species were not available, the universal Folmer primers, LCO1490 (5′ GGT CAA CAA ATC ATA AAG ATA TTG G 3′) and HC02198 (5′ TAA ACT TCA GGG TGA CCA AAA AAT CA 3′) (Folmer *et al.*, 1994) were used to amplify COI fragments which were sequenced. Products were aligned using the ClustalW utility implemented in BioEdit version 7.2.5 (Hall, 1999). Primers were designed and assessed using Primer3web version 4.1.0 (Untergasser *et al.*, 2012) and following best practice PCR primer design criteria (Taylor *et al.*, 2015). Following evaluation, the prey-specific primer sets were synthesized by Integrated DNA Technologies (Coralville, IA, USA). Primer sensitivity and specificity was empirically validated by PCR and gel-electrophoresis (Supplementary Table I). Amplification was accomplished using a GeneAmp 9 700 Thermocycler (Applied Biosystems, Foster City, CA, USA).

### qPCR assay and data analysis

Quantitative standards for qPCR were prepared by amplifying the mtCOI gene from each of the targeted prey species using the Folmer primer set, cloned into a plasmid and subsequently used as quantitative standards for the qPCR assay. Folmer PCR products were amplified by touchdown PCR: 5× [95°C (5 min), 94°C (30 s), 40°C (30 s), 72°C (1 min)] + 30× [94°C (30 s), 50°C (30 s), 72°C (1 min)] followed by 10 min at 72°C. Resulting products were extracted from agarose gels using the QIAquickGel Extraction Kit (Qiagen, Valencia, CA, USA) and cloned prior to sequencing using the Invitrogen™ TOPO TA Cloning Kit (Invitrogen, Waltham, MA, USA). DNA concentration of the resulting plasmids was quantified using a NanoDrop™ 2000c spectrophotometer (Thermo Scientific, Wilmington, DE, USA). Optimized annealing temperatures for each prey-specific qPCR assay were identified utilizing the Bio-Rad SsoFast™ EvaGreen® Supermix (Bio-Rad Laboratories, Hercules, CA, USA). qPCR reactions were conducted in 20 μL reaction volumes containing a final concentration of 1X SsoFast™ EvaGreen® Supermix, 0.3 μmol of each primer, and template concentrations of plasmid DNA (pDNA) containing a cloned copy of the target COI gene ranging from 10^1^ to 10^8^ copies per reaction.

Prey DNA concentrations derived from 123 female *Bythotrephes* specimens (n_Erlaufsee_ = 53; n_Mondsee_ = 70) were estimated by qPCR using each of the 7 prey-specific primer sets designed in this study (Table II). qPCR reactions were conducted in 20 μL reactions essentially as described above, except that template concentrations ranged from 0.23 ng to 8.14 ng target gDNA per reaction. Empirically optimized amplification annealing temperatures for each of the assays are reported in Table II. qPCR reaction conditions included an initial enzyme activation step at 95°C for 30 s followed by 40 cycles of denaturation (95°C, 5 s) and annealing/extension (Table II, 5 s). After cycling, product melt-temperatures were evaluated from 55°C to 95°C at 0.5°C increments for 5 s each.

The abundance of COI genes was estimated relative to standard curves prepared with quantified pDNA containing an insert of the target COI gene from the respective prey species (Supplementary Fig. 1). All qPCR reactions utilized a Bio-Rad CFX96 Real-Time PCR System. Samples, standards and no template controls were assayed in triplicate. For each triplicate, mean ct values were calculated. Samples were dismissed if two out of three replicates were below detection limits. Copy numbers were estimated from the ct values for each species, each qPCR run and associated standard curve. Mean efficiency (E) of all valid qPCR reactions was 105.5 (SD = 5.3), mean coefficient of determination (*R*^2^) was 0.98 (SD = 0.01), mean E and R^2^ for each prey species are summarized in Table III. Four qPCR runs were excluded from further analyses due to high (126.0%, 137.6%) or low (87.9%) amplification efficiencies and mean ct values of 37.1 and 36.5 for the nontemplate control.

**Table III:** Mean efficiency (E), coefficient of determination (R^2^) and standard deviation (SD) of qPCR runs for each prey species

*Species*	*Mean E*	*SD E*	*Mean R* ^2^	*SD R* ^2^
*M. leuckarti*	102.90	1.99	0.98	0.015
*E. gracilis*	108.93	5.48	0.98	0.005
*Leptodora kindtii*	109.82	3.02	0.99	0.004
*Diaphanosoma brachyurum*	98.63	1.80	0.99	0.002
*Cyclops sp. X*	102.80	0.80	0.99	0.001
*Bosmina*	103.30	4.78	0.99	0.006
*Daphnia*	105.45	4.83	0.97	0.014

Because *M. leuckarti* and *D. brachyurum* do not occur in Erlaufsee, specific primers for these species (Table II) were chosen to test for contamination/false positives in the MGCA by applying the primers to *Bythotrephes* sampled from this lake. Tests for *M. leuckarti* resulted in ten negatives and two signals (ct = 39.46 and ct = 36.77). Tests for *D. brachyurum* resulted in five negative specimens and five specimens with signals between ct 38.3 and 39.72. We therefore considered a cutoff at ct = 36 as an adequate condition for this data set. Subsequent results refer to counts with ct < 36.

Statistical analyses were conducted using R (R Core Team, 2019). Significant differences in diet between the lakes and between respective prey species were tested using Fisher’s exact test, based on counts of positive (ct < 36) versus the sum of inconclusive and negative signals (Table I), and Kruskal–Wallis test for differences in starting quantity (SQ) of positive counts (base R 3.6.1). Figures were plotted using the R packages ggplot2 (Wickham, 2016), scales (Wickham and Seidel, 2020), ggpubr (Kassambara, 2020), mdthemes (Neitmann, 2020) and dplyr (Wickham *et al.*, 2020).

## RESULTS

Prey-specific primer pairs were designed and validated (Table II, Supplementary Table I) and subsequently integrated into seven qPCR assays. These assays amplified COI mtDNA fragments ranging in size from 72 bp to 145 bp. All qPCR assays had a linear dynamic range from 10^1^ to 10^8^ target copies (Supplementary Fig. 1). Empirical testing by PCR including specimens of all species and populations included in this study demonstrated the expected specificity of the primers (Supplementary Table I). Subsequent results refer to positive signals with ct values <36.

**Fig. 1:**
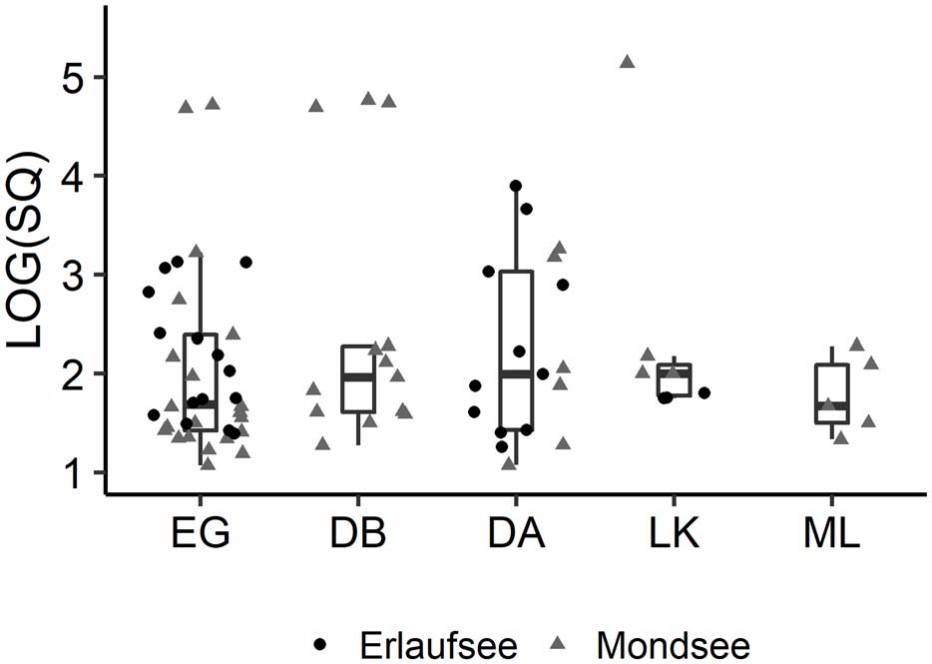
Starting quantity (SQ) per species demonstrating varying power of detected signals. Only signals with ct < 36 have been included [EG: *E. gracilis,* DB: *Diaphanosoma brachyurum*, DA: *Daphnia,* LK: *Leptodora kindtii,* ML: *M. leuckarti*].

**Fig. 2:**
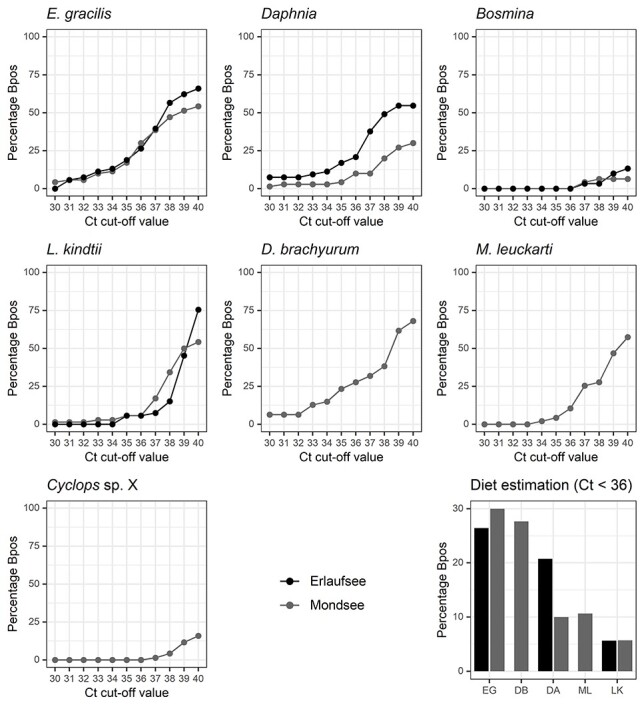
Results of the qPCR analysis depicting the percentage of *Bythotrephes* specimens with DNA signals (Bpos) depending on prey species and lake with different cycle threshold (ct) cut-off values. Total diet estimation depicts results with ct < 36 [EG: *E. gracilis*, DB: *Diaphanosoma brachyurum,* DA: *Daphnia,* ML: *M. leuckarti,* LK: *Leptodora kindtii*].

There were no significant differences in mean SQ values between the lakes (Kruskal–Wallis Test: *P* = 0.46, eta2[H] = 0.006) or the prey species (Kruskal–Wallis Test: *P* = 0.62, eta2[H] = 0.019) (Fig. 1). A small number of individuals showed orders of magnitude more prey signal than most specimens. *E. gracilis* and *D. brachyurum* were commonly detected (Fig. 2, Table I), demonstrating that the largest proportion of *Bythotrephes* diet in our study lakes seems to be comprised of these two species. A total number of 29 *Bythotrephes* specimens (41.4%) from Mondsee and 25 (47.2%) from Erlaufsee showed ct values below 36 for tested prey species. There were no significant differences in the frequency of prey detection between the two lakes (Fisher’s exact test: *P* = 0.58, odds ratio = 0.79, CI: 0.36, 1.73). There were no significant differences between Mondsee and Erlaufsee regarding the frequency of signal detection of *E. gracilis* (Fisher’s exact test: *P* = 0.69, odds ratio: 1.19, CI: 0.50, 2.89), *Daphnia* (Fisher’s exact test: *P* = 0.08, odds ratio: 0.38, CI: 0.12, 1.16) and *L. kindtii* (Fisher’s exact test: *P* = 1.0, odds ratio: 1.01, CI: 0.16, 7.21). In Mondsee, there were significant differences between the number of signals of *E. gracilis* and *Daphnia* (Fisher’s exact test: *P* = 0.005, odds ratio: 3.82, CI: 1.42, 11.54), *L. kindtii* (Fisher’s exact test: *P* = 0.0003, odds ratio: 6.98, CI: 2.16, 29.77) and *M. leuckarti* (Fisher’s exact test: *P* = 0.01, odds ratio: 3.56, CI: 1.17, 13.17). Similarly, significant differences could be detected between the frequencies of signals of *D. brachyurum* and *Daphnia* (Fisher’s exact test: *P* = 0.02, odds ratio: 3.4, CI: 1.14, 11.09) and *L. kindtii* (Fisher’s exact test: *P* = 0.002, odds ratio: 6.2, CI: 1.75, 28.13). but not *M. leuckarti* (Fisher’s exact test: *P* = 0.06, odds ratio: 3.17, CI: 0.94, 12.53). In Erlaufsee, differences regarding signal counts for *E. gracilis* and *L. kindtii* were significant (Fisher’s exact test: *P* = 0.007, odds ratio: 5.89, CI: 1.50, 34.16), but there was no significant difference between *E. gracilis* and *Daphnia* (Fisher’s exact test: *P =* 0.82, odds ratio: 1.22, CI: 0.46, 3.29). Eighteen specimens of *Bythotrephes* (n_Mondsee_ = 14; n_Erlaufsee_ = 4) tested positive for more than one prey species. No signals for *Bosmina* and *Cyclops sp.* X were observed.

**Fig. 3:**
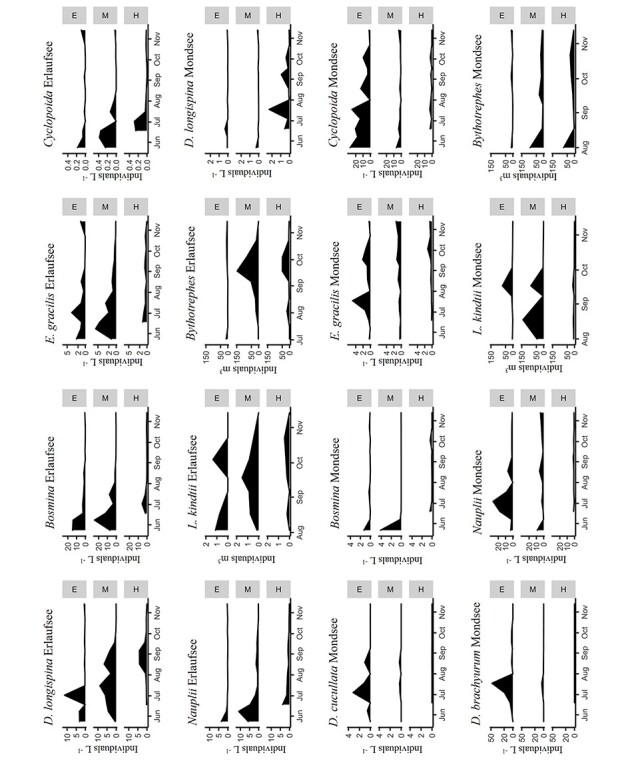
Seasonal abundance and vertical distribution of zooplankton in the two study lakes Erlaufsee and Mondsee. Counts for copepods refer to copepodites and adults. Cyclopoid copepods have been pooled. *M. leuckarti* was the dominant cyclopoid copepod in the epilimnion of Mondsee, other species are primarily found in the lower layers [E: epilimnion, M: metalimnion, H: hypolimnion].

*E. gracilis* was present in all lake layers of both study lakes throughout the sampling period (Fig. 3). *D. brachyurum* was the dominant cladoceran in Mondsee, peaking in mid-July and decreasing steeply in August. *Daphnia* was common in Erlaufsee, but comparatively rare in Mondsee. Similarly, *Bosmina* was common in early summer in Erlaufsee, but rarely present in Mondsee. *L. kindtii* was very abundant in Mondsee, but rare in Erlaufsee. In contrast to Erlaufsee, cyclopoid copepods were dominant in Mondsee with mostly *M. leuckarti* occupying the epilimnion.

## DISCUSSION

This study provides the first molecular *in situ* diet estimation of the predatory cladoceran *Bythotrephes* in its native range by MGCA using qPCR. Both cladoceran and copepod prey were consumed, confirming that *Bythotrephes* is a generalist predator. Main differences in the diet between the study lakes concern the occurrence of *D. brachyurum* and *M. leuckarti* in Mondsee and the absence of said species in Erlaufsee. *E. gracilis* and *D. brachyurum* were detected at a much higher frequency in the MGCA than other species (Fig. 2), suggesting some level of feeding selectivity.

To evade visual predators like fish, some zooplankton taxa are able to migrate into deep lake layers (Ringelberg, 2010). This behavior has also been observed for North American species as a response to the invasion of *Bythotrephes* (e.g. Pangle *et al.*, 2007; Bourdeau *et al.*, 2011). *E. gracilis* nauplii in Mondsee are reportedly distributed in the epilimnion, in stark contrast to nauplii of *Cyclops* (Nauwerck, 1988). Strict epilimnetic distribution has also been reported for *D. brachyurum* (Nauwerck, 1988; Gaviria-Melo *et al.*, 2005; Błędzki and Rybak, 2016), presumably benefitting the visual predator *Bythotrephes* (Muirhead and Sprules, 2003; Pangle and Peacor, 2009). Our data generally support these observations, especially *D. brachyurum* was very abundant in the epilimnion of Mondsee and *E. gracilis* was present in the upper layers of both study lakes throughout the sampling period (Fig. 3). Yet, vertical distribution alone might not explain choice of prey in all cases: In North America, the hypolimnetic calanoid *Senecella calanoides* decreased after *Bythotrephes* invasion, whereas *Limnocalanus macrurus* did not (Kerfoot *et al.*, 2016). A closely related North American species to *D. brachyurum*, *D. birgei*, was observed to decline in invaded lakes, but lab experiments could not observe evidence of predation impact on the genus *Diaphanosoma* (Jokela *et al.*, 2013). A slightly smaller proportion of observed *Daphnia* signals may imply more successful defense strategies. However, *Daphnia* were rare in Mondsee and quite abundant in Erlaufsee, which might explain the different proportion of positive signals between the lakes. It is unclear whether *Bythotrephes* in Mondsee prefer *D. longispina*, which is generally capable of vertical migration (Ringelberg, 2010), or *D. cucullata*, which exhibit epilimnetic distribution, but also conspicuous defense structures (Laforsch and Tollrian, 2004). In North America, different species of *Daphnia* were repeatedly described as preferred prey of *Bythotrephes* (e.g. Vanderploeg *et al.*, 1993; Pangle and Peacor, 2009; Kerfoot *et al.*, 2016). *M. leuckarti* also exhibits a primarily epilimnetic lifestyle (Nauwerck, 1988; Błędzki and Rybak, 2016; Nilssen and Wærvågen, 2000). In North America, *Mesocyclops edax* is a rare case of cyclopoid copepod reported to decline after *Bythotrephes* introduction (Barbiero and Tuchman, 2004; Kerfoot *et al.*, 2016). Similar to its close relative in Europe, *M. edax* is typically distributed above the thermocline (Marcogliese and Esch, 1992). *L. kindtii* was occasionally detected in *Bythotrephes* guts. Predation of North American *L. kindtii* has been observed in laboratory experiments (Branstrator, 1995). Interestingly, *L. kindtii’s* response to *Bythotrephes* has been described as neutral in Norway but negative in Canada (Kelly *et al.*, 2013).

Previous studies demonstrated noteworthy differences in the diet of *Bythotrephes* (see Introduction), which could only partially be explained by the chosen methods. Vanderploeg *et al.* (1993) showed spatial variations in the diet of *Bythotrephes* in Lake Huron. Whereas cladocerans were favored prey in one location and copepods were hardly eaten, in another location without its preferred prey, *Bythotrephes* exhibited a moderate clearance rate on nauplii. Similarly, we would expect seasonal diet variations in our study lakes, dependent on the abundance of different prey species. It is, e.g. conspicuous that the highest number of positives for *D. brachyurum* (Table I) was observed around the time of its peak abundance (Fig. 3). *D. brachyurum* was seasonally present in large numbers, constituting a notable source of prey. Future studies may focus on seasonal diet variations by substantially increasing the number of analyzed specimens and reporting how broad, specialized or dependent on prey abundances the diet is across the season. Here it would be advisable to sample both predators and prey from the whole water column to allow for correlating zooplankton abundances with observed diet proportions.

*Cyclops sp.* X and *Bosmina* could not be detected by qPCR given the applied cutoff. It should be noted that the detection of *Cyclops sp.* X in Mondsee represents its first Austrian record. Although predation of cyclopoid copepods by *Bythotrephes* has been previously observed (Vanderploeg *et al.*, 1993; Schulz and Yurista, 1995), it is generally considered uncommon, with some exceptions. For example, Kerfoot *et al.* (2016) observed the significant reduction of naupliar stages of copepods in invaded lakes. The case of *Bosmina* is particularly interesting, because *B.(E.) coregoni* itself has been introduced to the Great Lakes region (Mills *et al.*, 1993), where it has been reported to decline significantly in lakes where *Bythotrephes* has been introduced (Barbiero and Tuchman, 2004). One possible explanation for the lack of detection in the guts might be that in Mondsee, *Bosmina* was very rare and in Erlaufsee it was only common early in the season (Fig. 3). Further, Schulz and Yurista (1999) speculated that *Bosmina,* as well as copepod nauplii, could constitute a greater proportion of the diet of juvenile (i.e. instar I) *Bythotrephes*. We did not include the developmental status of *Bythotrephes* in this study, but instar II specimens were certainly most abundant in our samples (data not shown). Thus, samples analyzed may not reflect the full range of *Bythotrephes* diet across all instars. Future dietary studies should include, and distinguish between, all life stages of *Bythotrephes*. Whether *Bythotrephes* is the main reason for the observed changes in abundance and the pronounced vertical migration of some species in Mondsee and Erlaufsee is beyond the scope of this study and has to be analyzed carefully, with the inclusion of environmental lake data and study lakes without this predator (Pichler et al, in prep.).

There are several possible sources of bias that may affect the interpretation of MGCA results. Most notably, environmental contamination (Passmore *et al.*, 2006; King *et al.*, 2008), cannibalism (Pompanon *et al.*, 2012) and secondary predation (Sheppard *et al.*, 2005; King *et al.*, 2008) are relevant sources of error. Within this study, secondary predation might apply to potentially cannibalized conspecifics*,* copepods and *L. kindtii* in particular, while the remaining cladocerans are known to primarily feed on algae and bacteria. Zooplankton is sampled by net tows, concentrating many thousands of specimens, which might get entangled and leave traces of DNA on predators of interest as well. Besides separating and rinsing individual specimens, we attempted to diminish false positives by discarding late signals, knowingly accepting possible type II errors in the final diet estimation. Applying a cutoff and analyzing early qPCR signals is a common method to decrease the possible effect of contamination (see Introduction).

Detected signals differed regarding signal strength. In copepods, rapid digestion allows the detection of prey DNA up to 6 h after ingestion (Nejstgaard *et al.*, 2003; Durbin *et al.*, 2008). The rate of disappearance of PCR signals in these studies was correlated with estimates of gut evacuation rates, suggesting similar circumstances for *Bythotrephes*, which has an estimated gut passage time of 6–12 h (Yurista and Schulz, 1995). Detected signals most likely represent daytime feeding, because predation by *Bythotrephes* is light-dependent (Pangle and Peacor, 2009). Different sizes of prey species were not considered, because *Bythotrephes* is a sloppy feeder and does not ingest whole prey (Yurista *et al.*, 2010). Additionally, a positive signal does not indicate whether one or more specimens of the same prey species, small larvae or large adults have been captured, especially considering rapid digestion and that the time of ingestion is unknown. Particular cases of conspicuously high starting quantities (Fig. 1) could perhaps be explained by very recent consumption and/or predation of ovigerous females. The quantity of target detected is expected to scale with the number of eggs consumed (Weber and Lundgren, 2009). Cotterill *et al.* (2013) demonstrated that even a single developing oocyte may increase mitochondrial copy number on the order of a thousand-fold. Ovigerous females of *E. gracilis* had higher encounter and mortality rates in laboratory experiments with predatory fish than nonovigerous females, most likely due to their highly visible egg-clutch (Svensson, 1992). The same may be true for encounters with *Bythotrephes*. Although it is impossible to exclude methodological artifacts, it seems unlikely these would affect the major conclusions of this study.

## CONCLUSION

In this study, we estimated the diet of *Bythotrephes* in its native range. Based on 123 individuals collected from two Austrian lakes, the diet of *Bythotrephes* consisted of *E. gracilis*, *D. brachyurum, D. longispina* complex, *M. leuckarti* and *L. kindtii,* with the most commonly detected prey species being *E. gracilis*. The diet differs between the two studied lakes, likely due to differences in prey species composition. These observations support earlier conclusions that the spiny water flea is a generalist predator in both native and invaded ranges. Prey selection is therefore likely the result of differences in prey distribution and migration behavior in the water column. Future studies should aim to incorporate species-specific developmental status and corrections for breakdown of DNA in predator guts.

## Supplementary Material

bythotrephes_diet_supplement_fbab070
