# Supplementary material for: Application of species-specific primers to estimate the in situ diet of Bythotrephes [Cladocera, Onychopoda] in its native European range via molecular gut content analysis
Source: J Plankton Res. 2021 Oct 28;43(6):945–56. doi: 10.1093/plankt/fbab070 (PMC8632759; doi:10.1093/plankt/fbab070)
Supplement: bythotrephes_diet_supplement_fbab070 [file bythotrephes_diet_supplement_fbab070.docx]

**Application of species-specific primers to estimate the *in situ* diet of *Bythotrephes* [Cladocera, Onychopoda] in its native European range via Molecular Gut Content Analysis**

**SUPPLEMENT**

**Supplementary Table I**: Empirically determined specificity of COI-targeted species-specific qPCR assays [+: positive; -: negative; ML: *M. leuckarti*, EG: *E. gracilis*, CV: *C. vicinus,* CX: *Cyclops sp*. X, LK: *L. kindtii*, DB: *D. brachyurum,* BO: *Bosmina,* DA: *Daphnia*, MO: Mondsee, ER: Erlaufsee]

|  |  | PCR Amplification of Target Species | | | | | | |  | |  |
| --- | --- | --- | --- | --- | --- | --- | --- | --- | --- | --- | --- |
| Copepoda | **Prey species** | **ML** | **EG** | **CV** | **CX** | **LK** | **DB** | **BO** | | **DA** | |
|  | *M. leuckarti* MO | **+** | - | - | - | - | - | - | | - | |
|  | *E. gracilis* MO | - | **+** | - | - | - | - | - | | - | |
|  | *E. gracilis* ER | - | **+** | - | - | - | - | - | | - | |
|  | *C. vicinus* ER | - | - | **+** | - | - | - | - | | - | |
|  | *C. sp.* X MO | - | - | - | **+** | - | - | - | | - | |
| CLADOCERA | *L. kindtii* MO | - | - | - | - | **+** | - | - | | - | |
|  | *L. kindtii* ER | - | - | - | - | **+** | - | - | | - | |
|  | *D. brachyurum* MO | - | - | - | - | - | **+** | - | | - | |
|  | *Bosmina* MO | - | - | - | - | - | - | **+** | | - | |
|  | *Bosmina* ER | - | - | - | - | - | - | **+** | | - | |
|  | *D. longispina* MO | - | - | - | - | - | - | - | | **+** | |
|  | *D. longispina* ER | - | - | - | - | - | - | - | | **+** | |
|  | *D. cucullata* MO | - | - | - | - | - | - | - | | **+** | |
|  | *Bythotrephes* MO | - | - | - | - | - | - | - | | - | |
|  | *Bythotrephes* ER | - | - | - | - | - | - | - | | - | |


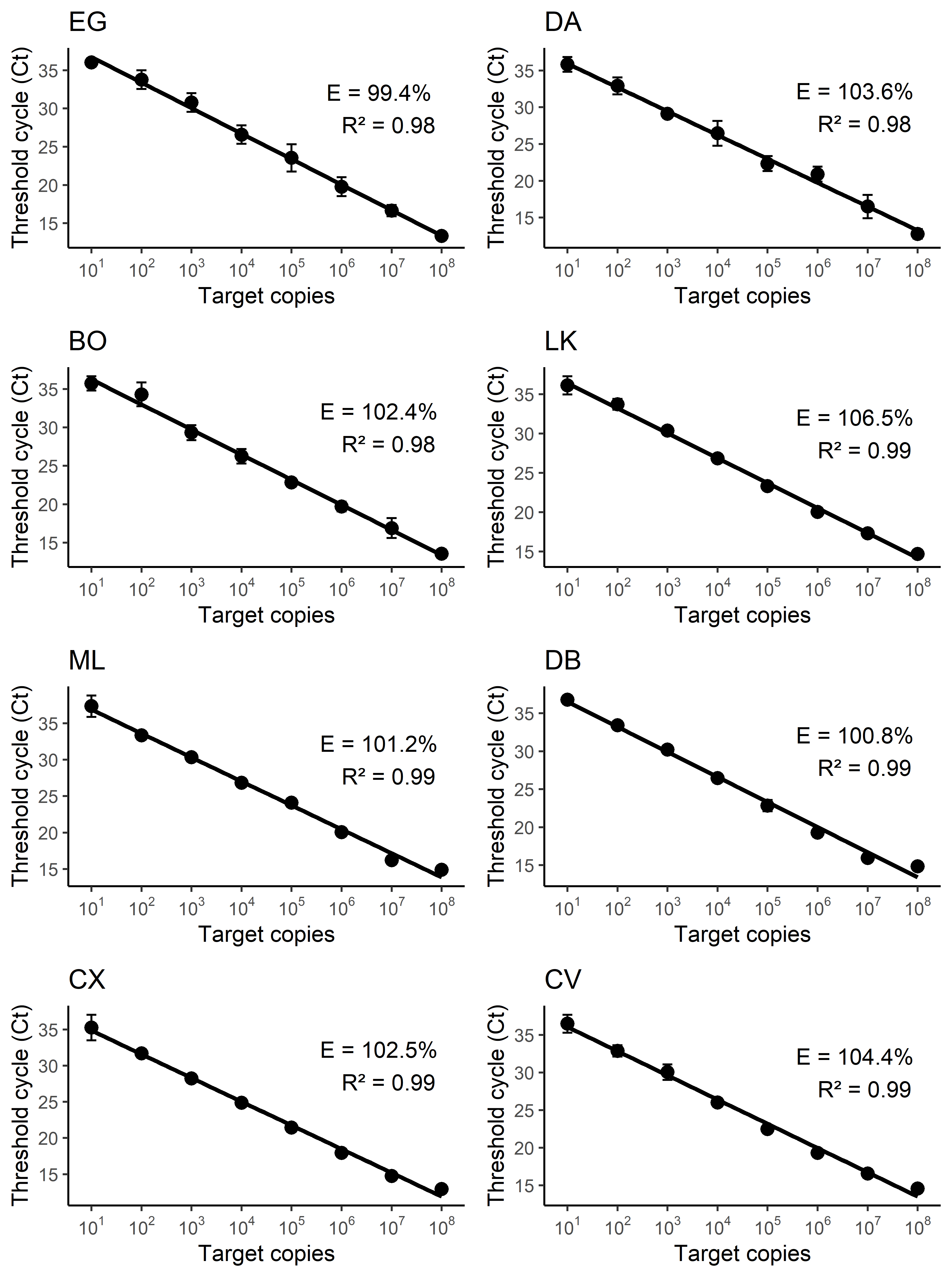


**Supplementary Fig. 1**. Examples of qPCR runs showing plasmid standards of cloned full-length COI fragments for each potential prey species (black solid circles). Error bars show standard deviation of threshold cycle. [EG: *E. gracilis*, DA: *Daphnia*, BO: *Bosmina*, LK: *L. kindtii*, ML: *M. leuckarti*, DB: *D. brachyurum*, CX: *Cyclops sp*. X, CV: *C. vicinus*, E= Efficiency, R^2^= coefficient of determination]
